# Society Meetings

**Published:** 1889-01

**Authors:** 


The Southern Surgical and Gynecological Association held a most successful meeting in Birmingham last month. Dr. Hunter McGuire, of Richmond, Va., was elected President for 1889—an earnest of continued good work.

